# Comparing Pre-Induction Ultrasound Parameters and the Bishop Score to Determine Whether Labor Induction Is Successful

**DOI:** 10.3390/medicina60071127

**Published:** 2024-07-12

**Authors:** Stevan Milatović, Anita Krsman, Branislava Baturan, Đorđe Dragutinović, Đorđe Ilić, Dragan Stajić

**Affiliations:** 1 Faculty of Medicine, University of Novi Sad, Hajduk Veljkova 3, 21000 Novi Sad, Serbia; stevan.milatovic@mf.uns.ac.rs (S.M.); branislava.baturan@mf.uns.ac.rs (B.B.); djordje.ilic@mf.uns.ac.rs (Đ.I.); dragan.stajic@mf.uns.ac.rs (D.S.); 2 Department of Obstetrics and Gynecology, University Clinical Center of Vojvodina, Branislava Ćosića 37, 21000 Novi Sad, Serbia; 3 Department of Computing and Control Engineering, Faculty of Technical Sciences, University of Novi Sad, Trg Dositeja Obradovića 6, 21000 Novi Sad, Serbia; djordje.dragutinovic@uns.ac.rs

**Keywords:** labor, induced, Bishop sore, ultrasound, prediction, cesarean section

## Abstract

*Background and Objectives*: The incidence of labor induction is steadily increasing worldwide. The main aim of this study was to evaluate the ultrasound parameters and their mutual correlation and to analyze the parameters’ predictive capability in assessing the success of labor induction. The secondary goal was to assess patients’ tolerability and acceptance of transvaginal ultrasound and digital gynecological examination. *Materials and Methods*: This prospective observational follow-up study included 252 women selected for labor induction. The transvaginal ultrasound examination measured the posterior cervical angle, cervical length, the length and width funneling of the cervix, the distance between the head of the fetus and the external uterine os, and the position of the fetal occiput. After the ultrasound, a digital vaginal examination was performed (according to the Bishop score), and the women were asked to rate their perception of pain for each procedure. *Results*: The most common indication for labor induction was post-term pregnancy (57.59%), and the most common method of labor induction was oxytocin with amniotomy (70%). The results showed that a significant independent prediction of vaginal delivery could be provided based on the Bishop score and cervical length. Other investigated ultrasound parameters, the length and width of the funneling of the cervix (*p* < 0.001), the fetal head stage (*p* < 0.001), and the size of the posterior cervical angle (*p* < 0.05), showed statistical significance in relation to the success of labor induction. Patients reported lower discomfort and pain during transvaginal ultrasound examination (mean score 2, IQR 3) compared to digital examination (mean score 5, IQR 4), with *p* < 0.001. *Conclusions*: The results imply that the assessment of ultrasound parameters before induction of labor is necessary to predict the outcome and reduce the possibility of complications. In terms of tolerability and choice by the patients, the transvaginal ultrasound examination was better rated than the vaginal gynecological examination.

## 1. Introduction

The incidence of induction of labor (IOL) as a standard obstetric procedure in obstetrics is approximately 20% [1,2]. Maternal and neonatal complications of this procedure include cesarean section, chorioamnionitis, postpartum hemorrhage, neonatal seizures, and the need for neonatal ICU admission [3]. Hence, there is a need for a good prediction of the labor induction outcome. Isolating pregnant women who are at high risk of failed induction of labor would lead to a reduction in maternal and neonatal morbidity and mortality. It would also result in optimized use of healthcare resources and reduced healthcare costs.

One of the common problems in the delivery department is differences among the cervical digital assessments by various medical team members. The Bishop score is commonly used to predict successful delivery, yet its sensitivity is 23–64% [4]. Recently, sonographic assessment of cervical measurements was shown to predict labor outcomes correctly [5,6,7]. Some literature data have shown a better predictive ability of transvaginal ultrasound (TVUS) parameters than the traditional Bishop score [8,9]. Although digital vaginal examination is the most common intervention that women have during pregnancy and childbirth, data on this type of examination of women are surprisingly scarce. There are documented studies that conclude vaginal exams are associated with pain and shame for patients and even claim that doctors use vaginal exams to demonstrate superiority over pregnant women [10,11]. Guided by the obtained results, some studies have recommended the routine use of ultrasound in predicting the outcome of labor induction and the first stage of labor [12,13,14].

This study aimed to evaluate the ultrasound parameters and their mutual correlation and analyze how good the parameters are as predictors in assessing the success of labor induction. We have selected the following ultrasound parameters: cervical length, size of the posterior angle of the cervix or its position, the length and width of funneling, distance of the fetal head from the external cervical os, and position of the fetal head. The secondary goal was to assess patients’ tolerability and acceptance of both procedures (transvaginal ultrasound and digital gynecological examination).

## 2. Materials and Methods

### 2.1. Study Design and Participants

This prospective observational follow-up study included 252 women admitted for labor induction at the Department of Obstetrics and Gynecology, University Clinical Centre of Vojvodina (Novi Sad, Serbia). All the women gave written informed consent to participate in the study, which the Clinical Center of Vojvodina Ethics Committee also approved (No 00-150; 11 February 2019) in accordance with the Helsinki Declaration.

The inclusion criteria were as follows: (1) singleton pregnancy, (2) cephalic presentation of the fetus, (3) gestational age 37–42 weeks of gestation, (4) vital pregnancy, (5) indications for induced labor, and (6) intact amniotic membranes.

Criteria for exclusion from the study: (1) previous pregnancy ended by caesarean section, (2) unknown information about the last menstruation and/or unknown probable due date, (3) allergy to prostaglandin preparations, (4) present congenital anomalies of the fetus, (5) twin pregnancies, (6) existing contraindication for vaginal birth, (7) earlier interventions on the cervix (conization, LLETZ), (8) previous operations on the uterus (operative hysteroscopy, myomectomy), (9) pregnant women in whom the presence of regular contractions was registered at the reception, (10) pregnant women who did not agree to be included in the research [15,16,17]. 

### 2.2. Procedures 

Gestational age was confirmed by the date of LMP and early first-trimester ultrasound. A non-stress test and ultrasound were performed to evaluate fetal well-being and growth. Ultrasound examination was performed using a Samsung Medison UGEO VS80A equipped with a transvaginal probe (2–10 MHz) and curved linear transducer (3–7.5 MHz), respecting the existing protocol (Fetal Medicine Foundation protocol) [18]. The transvaginal ultrasound examination measured the posterior cervical angle (PCA); cervical length (CL); the presence of cervical funneling and, if present, its length and width (FL, FW); the distance between the head of the fetus as the leading part and the external uterine os (FH, fetal head stage); and the position of the occiput of the fetus (transabdominal ultrasound examination) [19]. After emptying the bladder and placing the patient in the lithotomy position, the pregnant woman was examined with a transvaginal ultrasound probe. The endovaginal probe was introduced gently into the vagina in the direction of the anterior vaginal fornix. Cervical length measurement is the first observed parameter measured between the internal and external os according to the protocol of the Fetal Medicine Foundation [18]. For the cervical length measurement, the calipers were placed in a line between the internal and external cervical orifices. In the case of cervical tunneling, the length and width were measured. The cervical dilatation was the transverse measurement of the anechogenic line of the endocervical canal close to the fetal vertex [20]. Ultrasound-measured fetal head stage is defined as the distance between the lowest point of the fetal head (calvarium) and the external cervical os [20]. The posterior cervical angle was measured in a sagittal section as the angle between an imaginary line passing through the cervical canal and another tangential line at the junction of the posterior wall of the uterus and the internal orifice. In the case of a funnel-shaped or excessively curved cervix, the angle is estimated at the junction of the line that measures the length of the cervix and the back wall of the uterus [19]. When determining the position of the fetal head, the ultrasound abdominal probe was placed transversely in the suprapubic area of the pregnant woman. Landmarks used when defining the position of the fetal head were fetal orbits—posterior occipital presentation (OP; occiput–posterior position), central cerebral echo—transverse presentation (OT; occiput–transverse position), and cerebellum for anterior occipital presentation (OA; occiput–anterior position). The findings for each pregnant woman are incorporated into a circle (similar to a clock) with 24 divisions. The OA position included the occipital position between 09.30 and 02.30 h, transverse (OT) between 02.30 and 03.30 h or 08.30 and 09.30 h, and posterior occipital (OP) between 03.30 and 08.30 h [19]. The measurements were performed three times, and the smallest value was taken as the result. All the pregnant women underwent both procedures. The doctors performing the ultrasound measurements and vaginal examination (using the Bishop score) were blinded to each other’s findings. First, one doctor performed the ultrasound examination, and immediately before IOL, the Bishop score was assessed by another obstetrician who was blinded to the ultrasound measurements. After the examination, the women were asked to rate their perception of pain for each procedure (TVUS and vaginal examination). For that purpose, a 10-point visual analogue scale (VAS) was used, where a score of 0 meant “no pain” and a score of 10 meant “very painful”.

### 2.3. Cervical Ripening and Induction of Labor Protocol

Labor was induced according to standard protocol. Patients with an unfavorable cervix (Bishop score ≤ 6) received intracervical dinoprostone (0.5 mg/3 g; 2.5 mL gel). If the Bishop score was >6, labor was augmented using oxytocin at a starting dose of 6 mU/min with a 1-mU/min increase every 15 min until a regular uterine contraction was achieved (200–225 Montevideo Units or 3 contractions/10 min). A follow-up of labor progress was performed using a partogram. The study was designed in such a way that successful labor induction meant vaginal delivery within 24 h of the very beginning of labor induction. Unsuccessful IOL ended with surgery (cesarean section), i.e., interruption of induction of labor due to non-advancement of labor. Failed induction was defined as failure to achieve regular uterine contraction even after inserting 3 doses of intracervical dinoprostone gel at six-hour intervals and 12 h of oxytocin administration after rupture of the membranes [21]. Failure to progress was defined as no cervical dilatation during the active phase of the labor (≥4 cm) for the last two hours and no descent of the fetal head during the 2nd stage of labor for at least one hour despite adequate uterine contractions [22].

### 2.4. Data Analysis

Statistical analysis was performed in Python 3.7.3. (Python Software Foundation, Wilmington, DE, USA) and is presented in the tables. Continuous variables were expressed as mean ± standard deviation (SD). Categorical variables were described as count and percentage. Pearson’s chi-squared and Fisher’s exact tests were applied to check the significance of the results. Statistically significant results were those with *p* < 0.05. A one-way analysis of variance (ANOVA) was conducted to compare means between the groups.

## 3. Results

**Figure 1:**
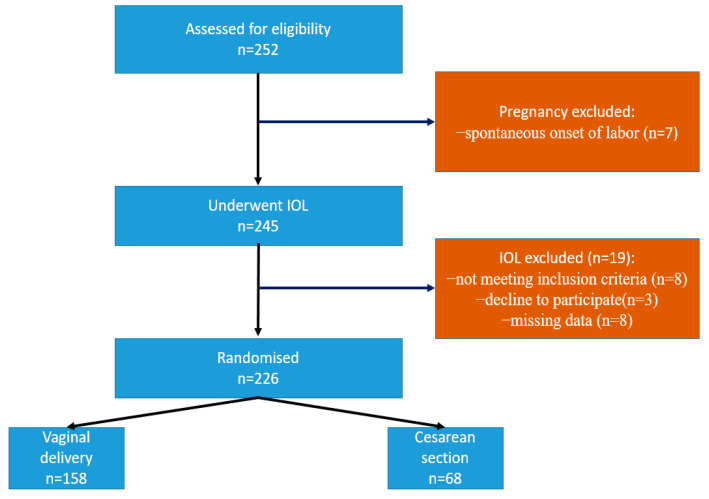
Flowchart: the process of inclusion, randomization, and exclusion of patients from the study.

A total of 252 women fulfilled the inclusion criteria and were enrolled in the study. Spontaneous onset of labor occurred in seven patients. Nineteen participants were excluded from the study because they underwent a cesarean delivery for unpredictable indications, e.g., fetal distress or for other reasons. So, 226 pregnancies were included in the analysis, as shown in Figure 1. The majority of pregnant women delivered vaginally (158) (Group A), while 68 pregnant women delivered by cesarean section (Group B) (Table 1). The mean age of study subjects was 29.40 ± 5.18 years. The mean gestational age was 40 weeks and 1 day at induction time. The mean BMI was 24.76 ± 3.8 kg/m^2^. There were no registered cases of maternal and neonatal death and no complications related to labor induction (hyperstimulation and rupture of the uterus).

Among the pregnant women whose induction of labor was completed successfully (Group A), most pregnant women (91–57.59%) had labor induced due to post-term pregnancy—which is the most common indication for the induction of labor. Other indications among pregnant women of both groups are shown in Table 2.

**Figure 2:**
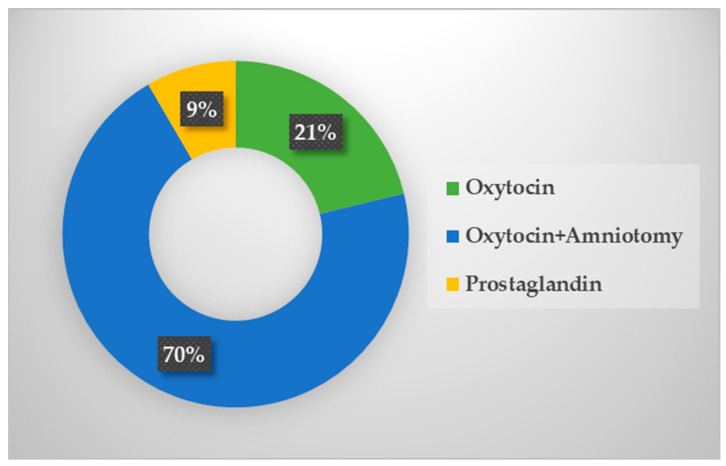
Methods of labor induction in the examined sample.

Out of the total number of pregnant women, 48 (21%) were induced only with oxytocin preparation. In 159 (70.35%) pregnant women, in addition to oxytocin, amniotomy was used as a method of labor induction. There were 19 (8.40%) women in which a prostaglandin preparation had to be applied before oxytocin in order to ripen the cervix (Figure 2).

The results analysis showed that the average Bishop score was 6.70 (SD 2.12; range 2–10), while the average length of the cervix, measured by transvaginal ultrasound, was 27.45 mm (SD 7.29; range 6–42). The pregnant women delivering vaginally had an ultrasound-measured length of the cervix of 25.14 ± 7.71 mm, while this parameter in the pregnant women delivering by cesarean section was 29.67 ± 6.09 mm (*p* < 0.001). The length and width of the funneling of the cervix (*p* < 0.001), the fetal head stage (*p* < 0.001), and the size of the posterior cervical angle (*p* < 0.05) showed statistical significance in relation to the success of labor induction. However, no statistical significance was shown for the position of the fetal occiput and the estimated body weight of the fetus in relation to the outcome of labor induction (Table 3).

Table 4 presents regression coefficients, odds ratios, and significance tests for each predictor, the Bishop score, and the tested ultrasound parameters. The cervical length was higher, and the Bishop score was lower in the cesarean section group compared to the vaginal delivery group. Multivariate regression analysis of the likelihood of vaginal delivery revealed a statistically significant association with the Bishop score (OR: 6.28, *p* < 0.001) and cervical length (OR: 0.38, *p* < 0.001) (Table 4). This analysis revealed that significant independent prediction of vaginal delivery could be provided by the Bishop score and cervical length but also by other ultrasound parameters such as length and width of cervical funneling, posterior cervical angle, and fetal head stage. The sensitivity and specificity of the Bishop score were 0.86 and 0.60, respectively. The sensitivity and specificity of cervical length were 0.83 and 0.66, respectively (Table 5). The cervical length and Bishop score cut-off points were 24 and 7, respectively. The findings of our study revealed that ultrasound measurement of cervical length is comparable to the Bishop score in predicting the outcome of labor induction. This study demonstrated that among pregnancies with over 37 weeks of gestation, a cervical length lower than 24 mm, as measured by transvaginal sonography, could predict that vaginal delivery is expected with a sensitivity of 93% and specificity of 86%. The same was found for the Bishop score, which was higher than 7, with a sensitivity of 90% and a specificity of 70%. The present study confirms that transvaginal sonographic cervical measurements (cervical length, the length and width of the funneling of the cervix, fetal head stage, and posterior cervical angle) are useful to predict the success of labor induction.

**Figure 3:**
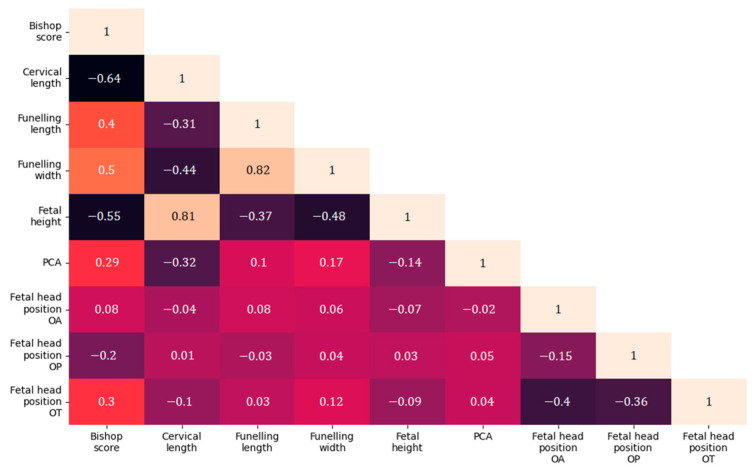
Correlation matrix of Bishop score and ultrasound parameters in relation to labor induction.

After the statistical analyses, a correlation analysis of the tested parameters was performed, shown in Figure 3.

The VAS showed a better tolerability of the transvaginal ultrasound examination than the digital examination. In addition, a lower sensation of discomfort and pain was registered in ultrasound (mean score 2, IQR 3) compared to the digital examination (mean score 5, IQR 4), *p* < 0.001.

## 4. Discussion

Labor induction is one of the most significant workloads for maternity services globally. The active management of labor has been proven effective in lowering neonatal and maternal morbidity by shortening the duration of labor. Several pharmaceutical and non-pharmaceutical approaches are currently being used in the active management of labor. There are various medications used for induction, including those commonly used for cervical ripening (prostaglandins) and oxytocin. Hyoscine butyl bromide (HBB), a drug that acts as a cervical spasmolytic agent, is an effective treatment for shortening the duration of the active phase of labor in primiparous and multiparous women [23]. Today’s research is aimed at discovering the most potent, natural, and least harmful method that could be used to end pregnancy at the right time in pregnant women whose risk for continued pregnancy exceeds that of giving birth itself. Despite numerous attempts, no ideal method has been found, so an individual approach is insisted upon to select the most correct one. In the future, pharmacogenomics may show that genetics can influence individual response and adverse reactions to various agents [24]. However, obstetricians still lack an optimal ultrasound-based diagnostic test to predict clinically relevant outcomes more accurately [25]. In relation to the investigated parameters, our results show that the length of the cervix and the tunneling, the posterior cervical angle, and the fetal head stage are parameters that can be used when evaluating the outcome of labor induction. Conflicting results have been reported when comparing the Bishop score and ultrasound measurements, such as the assessment of cervical length [26,27,28,29,30,31]. Strobel et al. [32] and Rozenberg et al. [33] demonstrated that the Bishop score and cervical length assessment (measured by ultrasound) are similarly accurate in predicting spontaneous labor. Strobel et al. [32] found that combining the Bishop score and cervical length is a better predictor than using each method alone. Keepanasseril et al. [34] reported a cut-off point of 30 mm for the cervical length, with a sensitivity of 85% and specificity of 91% for predicting vaginal delivery, so they favor cervicometry over the Bishop score in primiparous women for whom labor induction is indicated. Similarly, the findings of our study revealed that ultrasound measurement of cervical length is comparable to the Bishop score in predicting the outcome of labor induction. This study demonstrated that among pregnancies with over 37 weeks of gestation, a cervical length lower than 24 mm, as measured by transvaginal sonography, could predict that vaginal delivery is expected with a sensitivity of 93% and specificity of 86%. The same was found for the Bishop score, which was higher than 7, with a sensitivity of 90% and a specificity of 70%. Moreover, Tan et al. [5] demonstrated that ultrasound measurements of cervical length are similar to the Bishop score in predicting the necessity of cesarean section. Sharma et al. [35,36] found that out of 13 earlier studies on this topic, as many as 9 have shown that ultrasonographic cervical length is a better predictor of successful labor induction than the Bishop score. In a prospective study, Ben-Harush et al. [37] obtained a statistically significant correlation between sonographically measured cervical length and labor length. The group of pregnant women whose cervical length was <28 mm had a significantly shorter time interval from the beginning of labor induction to delivery. In a similar study (where cervical length was measured translabially), Khazardoost et al. [38] showed that greater cervical length carries a risk for operative termination of pregnancy. When cervical length is considered, among the women with cervical length > 2.8 cm, 48.9% had successful induction, whereas in women with cervical length ≤ 2.8 cm, 84% had successful induction [39]. In another two recent studies conducted by Shekhawat and Ibrahim, cervical length by TVUS is a useful and independent predictor of successful labor induction. When CL < 3.5 cm, 88% (66/75) delivered vaginally and when CL > 3.5 cm, only 11.42% (4/35) delivered vaginally [40,41]. A study by Kehlia and colleagues almost showed similar results [42]. On the other hand, we also have available studies that are inconsistent with the above results, where there was no statistical significance between ultrasound-measured cervical length and length of labor, nor the success of labor induction and the length of the latent phase of labor [26,33,43]. Hatfield et al. [44] also concluded that ultrasonographically measured cervical length was not a reliable predictor of delivery outcome. Studies have shown that the posterior cervical angle can be used as a predictor of vaginal delivery [45]. Studies by Rane et al. [19] and Kamel et al. [25] favor cervical length and size of the posterior cervical angle as ultrasound predictors of the outcome of labor induction, where PCA greater than 120° is associated with a positive response to IOL within 24 h. Ultrasonographic determination of the fetal head stage is performed by measuring the distance between the lowest point of the fetal head (calvarium) and the external cervical os. The height of the leading part of the fetus (fetal head stage) can be considered one of the parameters for predicting the success of labor induction [19,30], which aligns with our results (Table 3 and Table 4). Clinical studies have also shown that the incidence of cesarean section is more frequent in the case of reverse rotation of the fetal head (occiput posterior) [25,46,47]. As a result, fetal occipito-posterior position is associated with a higher possibility of cesarean section. Additionally, when the baby is in the occipito-posterior position, vaginal delivery could be complicated by perineal tears or an extension of an episiotomy [48]. Given that the position of the fetal occiput can easily be determined by transabdominal ultrasound examination, it is recommended that this parameter is included in the routine ultrasound examination of a pregnant woman when deciding on induction of labor [47]. However, the results of our research regarding the position of the occiput as a possible ultrasonographic predictor of the outcome of labor induction coincide with the systematic review conducted by Verhoeven et al. [48], who concluded that ultrasound evaluation of the occipital position of the fetal head should not be used in predicting the mode of delivery. In addition to the position of the occiput of the fetus, ultrasound-estimated fetal body mass also did not show statistical significance in relation to IOL outcome (Table 3).

The ultrasound examination has numerous advantages, such as enabling a more objective, accurate assessment of the cervix and the reproducibility of the examination, as well as the possibility of imaging the desired parameters. Moreover, transvaginal ultrasound has the additional advantage of visualizing the whole length of the cervix while assessing the internal cervical os for the presence of funneling, which would be difficult with a digital vaginal examination. As we must not neglect the satisfaction of patients, it should be noted that women tolerate transvaginal ultrasound examination better compared to digital vaginal examination, expressing significantly less discomfort, as shown by Abdullah et al. [49] (mean score 2, IQR 3 vs. mean score 5, IQR 4) and confirmed in our study. Tan et al. [5] and Gunes et al. [50] found a positive association between discomfort during vaginal examination and emotional violence, as well as post-traumatic stress disorder. Therefore, a transvaginal ultrasound examination is a tool that should be favored because the patients tolerate it better. It is associated with a lower feeling of pain and discomfort compared to the digital examination, which could undoubtedly contribute to the reduced anxiety of the patients and an increase in the positive experience of childbirth.

Many studies describe new parameters for a better assessment of cervical changes during pregnancy, which shows a great interest in and need for the scientific community to find an objective, reliable and applicable evaluation method and predictor of the success of labor induction. Despite efforts, the ideal parameter for predicting the success of labor induction has yet to be found.

**Limitations and advantages of the study**. The present study has potential limitations that should be overcome in further research. First, different induction methods may have an effect on the duration and outcome of labor. Therefore, an assessment of the IOL’s success with other methods not used at the Department of Obstetrics and Gynecology in Novi Sad (e.g., Foley catheter or misoprostol) is needed. Second, besides cervical status, the mode of delivery is influenced by various clinical factors such as gestational age, parity, and maternal BMI [4,6,51,52,53,54,55,56]. In addition to the above, it should be borne in mind that childbirth is a complex physiological process and that the clinical characteristics of the pregnant woman (age, parity, body mass index), as well as the fetus with its own characteristics (gestational age, fetal body weight), can influence the outcome of labor induction. Third, although we included fetal station and rotation in our study, the occiput–spine angle and flexion of the fetal head were not considered as a possible and additional variable for labor progress. A further weakness was our inability to analyze in more detail the patients who delivered by caesarean section. Patients who underwent caesarean section due to fetal distress and potential placental insufficiency were excluded from the sample. This came from the way women were recruited into the study but also represents the reality among women whose labor was induced. Exclusion of these patients probably led to selection bias. The relatively limited sample size was a weakness, especially regarding the operative delivery numbers. The success of TVUS in predicting the outcome of labor induction should be further determined by external validation through multicenter studies on a larger sample of patients. In that case, it is necessary to hire a larger number of researchers, which represents the weak point of the external validation as it could increase the variation among TVUS findings. It has, however, been demonstrated that the skills needed for examining women in labor with ultrasound are easily obtained [57] and have a shorter learning curve than vaginal examination skills [33]. The varying degree of experience in clinical vaginal examinations by the labor ward staff might likewise be considered a weakness. For this reason, the method of data collection is explained in detail throughout the study, with a special emphasis on TVUS and the measurement of the mentioned parameters.

On the other hand, the study has several advantages. The main strength of our study was its prospective design, performing the analysis with the objective of prediction. The varying degree of experience in clinical vaginal examinations by the labor ward staff might likewise be considered a weakness and we could not influence that. As for TVUS, interobserver variability was eliminated because ultrasound examination was performed by the same investigator. In particular, it should be noted that the clinicians managing the induction and delivery process were blinded to the initial investigator assessment, and all patients were followed consistently until delivery.

Nowadays, the success of labor induction becomes a question of extreme importance, because it is not only a question of the lowest possible mortality and morbidity of the mother and fetus but also the safety, efficiency, and duration of certain methods of labor induction, as well as the mother’s satisfaction. So the research on adequate methods of labor induction, as well as the prediction of the success of labor induction, will continue. Although there are data in the literature about elastography of the cervix and labor induction [58,59], we did not use this diagnostic method in the research. The subject of future investigations should also include some newer parameters such as sonographic evaluation of the consistency of the cervix or the cervical texture in order to predict the occurrence of spontaneous labor but also the outcome of labor induction. As a step forward, in future research it is planned to develop a model for predicting the success of labor induction based on machine learning. The presented study is part of a wider project that aims to create a software application that can be used as a data collector but which would allow continuous improvement of data sets and the accuracy of the model.

## 5. Conclusions

Ultrasound is an accessible, objective, easy-to-perform, and reliable predictor of IOL outcomes. Therefore, an ultrasound examination with the specified parameters should be performed before the decision on labor induction. In addition, this study has shown that patients tolerate this type of examination better than a vaginal gynecological examination. More extensive trials with the same methodology should be conducted to demonstrate the external validity of our findings and to find new ultrasound parameters to test the outcome of labor induction to reduce complications and perinatal morbidity and mortality.

## Figures and Tables

**Table 1:** Mode of birth and parity in the examined sample.

Mode of Birth	N (%)	Parity
Vaginal delivery	158 (69.9%)	Primipara 79 (50%)
		Multipara 79 (50%)
Cesarean section	68 (30.1%)	Primipara 52 (76.47%)
		Multipara 16 (23.52%)

(N) Number of patients in the sample, (%) percentage of participants.

**Table 2:** Presentation of indications for labor induction.

Indication for Labor Induction	Vaginal Delivery, N (%)	Cesarean Section, N (%)	Total, N (%)	*p-*Value
Post-term pregnancy	91 (57.59)	33 (48.53)	124 (54.87)	<0.001
Hypertensive syndrome in pregnancy	25 (15.82)	23 (33.82)	48 (21.24)	0.02
Oligohydramnios	18 (11.39)	2 (2.94)	20 (8.85)	0.006
Favorable obstetric finding	9 (5.70)	7 (10.29)	16 (7.08)	0.09
Other	11 (6.96)	3 (4.41)	14 (6.19)	0.038
IUGR	3 (1.90)	0.0	3 (1.33)	0.99
Cholestasis obstetrics	1 (0.63)	0.0	1 (0.44)	1.00

(N) Number of patients in the sample, (IUGR) intrauterine growth restriction, (“other”) PROM, diabetes mellitus, reduced fetal movements, (%) percentage of participants, (*p*) testing probability.

**Table 3:** Ultrasound parameters in relation to labor induction.

Characteristic	Successful Induction (Mean ± SD)	Cesarean Section (Mean ± SD)	*p-*Value
Cervical length [mm]	25.14 ± 7.71	29.67 ± 6.09	<0.001
Funneling length [mm]	6.81 ± 5.16	4.53 ± 6.05	<0.001
Funneling width [mm]	3.72 ± 2.63	1.91 ± 2.37	<0.001
Posterior cervical angle <120, n (%) ≥120, n (%)	116.16 ± 16.17 95 (60.13%) 63 (39.87%)	109.69 ± 19.09 40 (58.82%) 28 (41.18%)	0.027
Fetal head stage [mm]	32.00 ± 6.54	36.38 ± 5.52	<0.001
Position of the fetal head OP, n (%) OA, n (%) OT, n (%)	11 (6.96%) 33 (20.89%) 114 (72.15%)	19 (27.94%) 9 (13.24%) 40 (58.82%)	0.259
Estimated fetal weight (gram)	3536.36 ± 515.58	3460.87 ± 498.67	0.292
Bishop score	7.66 ± 1.82	5.77 ± 1.97	<0.001

(OA) occiput anterior, (OT) occiput transverse, (OP) occiput posterior, (%) percentage of participants, (*p*) testing probability.

**Table 4:** Display of significance tests for the examined parameters.

Characteristic	SE	*p*-Value	OR	95% CI
Bishop score	0.15	<0.001	6.28	(6.49–7.08)
Cervical length	0.49	<0.001	0.38	(26.49–28.41)
Funneling length	0.38	<0.001	2.83	(4.89–6.40)
Funneling width	0.18	<0.001	3.91	(2.44–3.15)
Fetal head stage	0.43	<0.001	0.27	(33.39–35.09)
Posterior cervical length	1.20	<0.05	1.52	(110.48–115.23)

(SE) standard error, (95% CI) confidence interval, (*p*) testing probability, (OR) odds ratio.

**Table 5:** Diagnostic characteristics of Bishop score and ultrasound parameters in predicting successful induction of labor.

Characteristic	Cut-Off	Sensitivity	Specificity	LR+	LR−	AUC
Bishop score	7	0.86	0.60	2.14	0.23	0.73
Cervical length	24	0.83	0.66	2.42	0.26	0.74
Funneling length	10	0.69	0.51	1.41	0.51	0.60
Funneling width	6	0.68	0.57	1.53	0.60	0.61
Posterior cervical angle	110	0.41	0.67	1.27	0.87	0.60
Fetal head stage	30	0.43	0.83	2.5	0.69	0.63

(LR+) likelihood ratio positive, (LR−) likelihood ratio negative, (AUC) area under the curve.

## Data Availability

All data involved in this work will be made available by the corresponding author upon request (anita.krsman@mf.uns.ac.rs; anitaanaaleksic@gmail.com).
